# Perceived Stigma in Health Care Settings and the Physical and Mental Health of People of Color in the United States

**DOI:** 10.1089/heq.2018.0079

**Published:** 2019-03-21

**Authors:** Henna Budhwani, Prabal De

**Affiliations:** ^1^ Department of Health Care Organization and Policy, University of Alabama at Birmingham, Birmingham, Alabama.; ^2^ Department of Economics, Colin Powell School, City College, New York, New York.; ^3^ Department of Economics, The Graduate Center, CUNY, New York, New York.

**Keywords:** disparities, mental health, race, stigma

## Abstract

**Purpose:** Addressing perceived and enacted stigma in clinical settings is critical to ensuring delivery of high-quality patient-centered care, reducing health disparities, and improving population health outcomes.

**Methods:** Data from the Behavioral Risk Factor Surveillance System's (2012–2014) Reaction to Race module were analyzed to test the hypothesis that perceived stigma in health care settings would be associated with poorer physical and mental health. Poor health was measured by (1) the number of days the respondent was physically or mentally ill over the past month and (2) depressive disorder diagnosis. Multivariate linear and logistic regression models were employed.

**Results:** Effects of stigma on physical and mental health were significant. Perceived stigma was associated with additional 2.79 poor physical health days (β=2.79, confidence interval [CI]=1.84–3.75) and 2.92 more days of poor mental health (β=2.92, CI=1.97–3.86). Moreover, perceived stigma in health care settings was associated with 61% higher odds of reporting a depressive disorder (adjusted odds ratio=1.61, CI=1.29–2.00). Among other findings, individuals who were married, younger, had higher income, had college degrees, and were employed reported significantly fewer poor physical and mental health days and had lower odds of self-reported depressive disorder.

**Conclusions:** Reducing stigma against people of color in health care settings (environments that should be pro-patient) must be a top priority for population health scholars and clinicians. Reducing perceived stigma in clinical settings may produce better mental and physical health outcomes in minority patients thereby reducing health disparities. In addition, fewer days lost to poor health could positively influence the health care system by decreasing utilization and may improve economic productivity through increasing days of good health.

## Introduction

The United States is undergoing a rapid demographic transition with about a third of the American population currently identifying as a racial or ethnic minority or person/people of color.^1,2^ This is a significant consideration when selecting evidence-based techniques to promote culturally appropriate patient centered care,^3–7^ because these groups often have unique health care needs and may interact with the health care system in ways that are unfamiliar to clinical providers, particularly providers who are not people of color themselves.^3–7^ Since these patients are physically identifiable as “different,” they are susceptible to race-based stigma and discrimination both in and out of the health care system.^7–9^

Stigma occurs when a group of individuals is devalued due to attributes deemed as undesirable.^10,11^ Stigma mechanisms—enacted, anticipated (or perceived), and internalized—are routinely examined in public health and health services research.^10–12^ Enacted stigma refers to actual experiences of discrimination; anticipated or perceived stigma represents expectation of repercussions; and internalized stigma is the acceptance of negative societal characterizations, labels, and perceptions about—in this study—people of color. Stigma, in all its forms, has been linked to negative patient health outcomes (e.g., poor medication adherence, missed doctor visits) across a range of illnesses.^11–15^ Stigmatizing experiences may occur in the greater community and in environments that should be stigma-free such as a patient's clinic.^16^ Ample studies have been conducted examining health care-based stigma and physical health status,^17–24^ with far fewer health disparity studies considering effects on both physical and mental health. Considering the importance of reducing health disparities in an effort to enhance overall population health and the growing attention being given to mental health, we test the hypothesis that perceived stigma in health care settings, attributed to being a racial or ethnic minority, will be associated with poorer physical health and worse mental health.

Previous studies consistently find that people of color experience stigma and discrimination across the community—where they work, where they live, and when they receive health care; these stigmatizing experiences have significant negative implications for well-being.^12–15,18–24^ Furthermore, the influence of demographics, namely age, gender, marital status, and geographic location, along with education and income—on experiences of stigma and health status cannot be overstated.^25–28^ Of particular importance is intersectional stigma (and discrimination), that is experienced when a person of color holds multiple stigmatizing roles such as being a woman and being Hispanic or being elderly and African American.^28–32^ Essentially, stigma and discrimination, when intersectional, may be exacerbated leading to amplified negative consequences.^28–32^ Conversely, being married thereby having access to ongoing social support may be protective, buffering the individual from negative health consequences.^33,34^ Increased income may allow the individual to change medical practices or work environments, if experiencing stigma. Therefore, higher income is typically a protective force. These prior study findings necessitate the inclusion of demographic measures in health services and public health research, especially when the goal is to reduce disparities by understanding the effects of countervailing forces.

## Methods

### Data sources

We analyze data from the Behavioral Risk Factor Surveillance System (BRFSS),^35^ an annual survey that collects data on public and individual health issues, outcomes, risk factors, and behaviors. Until recently, the survey has been unique in implementing its Reactions to Race module that was designed by the Centers for Disease Control and Prevention (CDC) Measures of Racism Working Group. This module asks respondents about their perceptions and experiences of reactions to race at work or in a health care setting. Unfortunately, over the years, the number of states implementing the module has dwindled, and since 2015, such variables are not found in the public data, making the 2014 BRFSS data analyzed in our study the latest available data. We pool data from 2012 to 2014 as BRFSS changed the survey methodology in 2011, making data from before and after 2011 noncomparable.

### Stigma measure

Perceived stigma was measured through the question: “When seeking health care past 12 months, was experience worse, same, better than people of other races?” The variable “Perceived Stigma: Health Care” was assigned 1=“Worse than other races” and 0=“Same as other races” or “Better than other races.”

### Outcome measures

Our three outcome or dependent variables pertain to physical health status, mental health status, and diagnosis for depressive disorder. For the self-reported physical health status, we use the survey responses to the questions: “Now thinking about your physical health, which includes physical illness and injury, for how many days during the past 30 days was your physical health not good?.” Accordingly, this is a continuous variable that assumes value between 0 and 30. There are two mental health measures in the BRFSS during the period 2012–2014. First, similar to physical health, respondents were asked “Now thinking about your mental health, which includes stress, depression, and problems with emotions, for how many days during the past 30 days was your mental health not good? Coding for this variable was identical to that of the physical health variable. In addition, the survey asked the question “Have you ever been told you had a depressive disorder?” This variable is coded as a binary variable, the value 1 being assigned to the answer “yes,” 0 otherwise.

### Statistical analyses

For the continuous dependent variables described above, we used multivariate linear regression models to analyze the association between perceived stigma and poor physical and mental health outcomes. For the depressive disorder measure, logistic regressions were used. All regression models control for the demographic and health care access factors found in the literature—gender, age, race and ethnicity, income, and health insurance coverage. We also control for key health services indicators, namely existence of a primary care (or personal) physician and medical cost being prohibitively high in the past year to see a doctor. Our analysis accounts for the complex survey design of the BRFSS, and all estimates are weighted to obtain appropriate standard errors. Coefficients are weighted via the final weight provided in the data set to represent the corresponding population. Regression diagnostics were conducted to identify multicollinearity using a variance inflation factor (VIF). All mean VIF values were less than 2.1, indicating that the models do not suffer from potential multicollinearity problems. Throughout the analysis, the responses of “Don't Know” and “Refused to Answer” were coded as missing observations. Given the size of the BRFSS sample, such practices do not affect the analyses in any significant way.

### Ethics approval

Studies that analyze data from the publically available BRFSS dataset are exempted from additional Institutional Review Board evaluation by the University of Alabama at Birmingham (UAB).

## Results

Table 1 provides descriptive statistics of the sample. Employment and health insurance coverage rates are similar for all racial and ethnic groups. Both Hispanics and African Americans report medical care as being too costly at higher rates (15%) than whites (7%) and Asian Americans (8%). Asian Americans report having a primary care physician at a much lower rate than all other race and ethnicity groups. The percentage of respondents who believed that they had experienced stigma due to their race in health care settings was higher for all racial and ethnic categories as compared to nonminority respondents. African Americans report the highest levels of stigma in health care settings, and the average numbers of reported bad physical and mental health days were higher for Hispanics and African Americans compared with whites and Asian Americans, arguably making African Americans more socially vulnerable compared with other minority peer groups.

**Table 1.** Health Outcomes, Perceived Stigma, and Sociodemographic Characteristics Across Four Racial and Ethnic Classifications–Behavioral Risk Factor Surveillance System Data 2012–2014

Variable	White only	African American only	Hispanic	Asian only
	% (or Mean)	% (or Mean)	% (or Mean)	% (or Mean)
Health outcomes				
Number of days respondent's physical health bad in 30 days	3.61	4.38	4.58	4.36
Number of days respondent's mental health bad in 30 days	3.23	4.49	4.63	3.90
Equal to 1 if ever told you had a depressive disorder	0.22	0.15	0.21	0.20
Stigma (%)				
Equal to 1 if perceived stigma in health care	2.06	8.31	7.12	3.70
Demographic variables (%)				
Equal to 1 if female	51.01	57.77	53.88	48.59
Equal to 1 if married	59.86	32.33	30.60	48.48
Equal to 1 if college graduate	31.20	16.49	11.91	11.80
Equal to 1 if employed	62.95	55.32	52.15	59.58
Age (%)				
18–24	4.55	5.15	8.47	8.48
25–34	9.68	14.16	15.34	14.82
35–44	12.39	16.48	18.48	17.39
45–54	18.79	18.27	23.52	20.15
55–64	24.98	23.25	17.60	21.04
65 or older	29.62	22.69	16.58	18.11
Health Care				
Equal to 1 if has health care coverage	92.35	79.79	86.69	73.64
Equal to 1 if could not see doctor because of cost	10.70	22.26	15.12	24.84
Equal to 1 if has a primary care physician	79.31	75.66	61.90	64.28
Observations	32,020	2,895	1,369	4,904

Tables 2–4 report results from regression analyses. In all the tables, column [1] and column [2] present results from models without and with control variables, respectively. In Table 2, results on the associations between stigma and physical health status with and without controlling for race and ethnicity, gender, age, education, employment, health care coverage, affordability of medical care, primary care physician, and income are reported.

**Table 2.** Associations of Perceived Stigma with Physical Health–Ordinary Least Square Regressions Using Behavioral Risk Factor Surveillance System Data 2012–2014

Variables	(1)	(2)	(3)	(4)
	Coeff.	95% CI	Coeff.	95% CI
Stigma in health care	5.09^***^	4.01–6.18	2.79^***^	1.84 to 3.75
Female			−0.52^***^	−0.78 to −0.25
Race/ethnicity (Ref: White)				
African American			−0.78^***^	−1.22 to −0.34
Hispanic			−0.65	−1.61 to 0.31
Asian			−0.08	−0.60 to 0.43
Other			−0.73^***^	−1.20 to −0.25
Age (Ref: 18–24)				
25–34			1.66^***^	1.24 to 2.08
35–44			2.57^***^	2.11 to 3.02
45–54			3.48^***^	3.00 to 3.95
55–64			4.02^***^	3.44 to 4.59
65 or older			1.70^***^	1.22 to 2.18
Married			−0.44^**^	−0.79 to −0.09
College graduate			−0.64^***^	−0.89 to −0.39
Employed			−3.59^***^	−3.96 to −3.23
Has health care coverage			1.36^***^	0.75 to 1.98
Could not see doctor because of cost			2.78^***^	2.23 to 3.33
Has a primary care physician			1.12^***^	0.78 to 1.46
Income (Ref: less than $10,000)				
$10,000 to less than $15,000			0.11	−1.16 to 1.38
$15,000 to less than $20,000			−0.51	−1.41 to 0.39
$20,000 to less than $25,000			−1.63^***^	−2.45 to −0.82
$25,000 to less than $35,000			−2.24^***^	−3.04 to −1.43
$35,000 to less than $50,000			−2.27^***^	−3.07 to −1.46
$50,000 to less than $75,000			−2.88^***^	−3.65 to −2.11
$75,000 or more			−3.44^***^	−4.20 to −2.67
Constant	3.65^***^	3.51 to 3.79	4.11^***^	3.24 to 4.99
Observations	60,491		41,775	

Dependent variable: no. *of bad physical health days* in 30 days before interview. Stigma in health care=1 if answer to the question “When seeking health care past 12 months, was experience worse, same, better than people of other races?” is “Worse than other races” and 0 if “Same as other races” or “Better than other races.” All estimates are weighted by the weights provided by the BRFSS. Regression diagnostics were conducted to identify multicollinearity using a VIF to indicate a potential problem. All mean VIF values were less than 2.1.

^***^ *p*<0.01.

CI, confidence interval; Coeff., coefficients from linear ordinary least square regressions; VIF, variance inflation factor.

**Table 3.** Associations of Perceived Stigma with Mental Health–Ordinary Least Square Regressions Using Behavioral Risk Factor Surveillance System Data 2012–2014

Variables	(1)	(2)	(3)	(4)
	Coefficient	95% CI	Coefficient	95% CI
Stigma in health care	5.18^***^	4.67 to 5.69	2.92^***^	1.97 to 3.86
Female			0.34^*^	−0.02 to 0.69
Race/ethnicity (Ref: White)				
African American			−0.96^***^	−1.48 to −0.45
Hispanic			−0.94^*^	−2.04 to 0.17
Asian			−0.93^***^	−1.59 to −0.26
Other			0.01	−1.35 to 1.36
Age (Ref: 18–24)				
25–34			1.07^***^	0.43 to 1.72
35–44			1.69^***^	0.95 to 2.44
45–54			1.14^***^	0.57 to 1.71
55–64			0.46	−0.09 to 1.00
65 or older			−2.50^***^	−3.11 to −1.88
Married			−0.83^***^	−1.22 to −0.45
College graduate			−0.59^***^	−0.84 to −0.33
Employed			−2.69^***^	−3.21 to −2.18
Has health care coverage			1.43^***^	0.85 to 2.01
Could not see doctor because of cost			2.85^***^	2.30 to 3.40
Has a primary care physician			0.44^**^	0.04 to 0.83
Income (Ref: less than $10,000)				
$10,000 to less than $15,000			−1.64^***^	−2.79 to −0.49
$15,000 to less than $20,000			−1.49^**^	−2.87 to −0.11
$20,000 to less than $25,000			−3.09^***^	−4.07 to −2.11
$25,000 to less than $35,000			−3.39^***^	−4.42 to −2.35
$35,000 to less than $50,000			−3.65^***^	−4.60 to −2.69
$50,000 to less than $75,000			−3.91^***^	−4.90 to −2.92
$75,000 or more			−4.51^***^	−5.47 to −3.55
Constant	2.96^***^	2.90 to 3.02	6.78^***^	5.70 to 7.87
Observations	60,676		41,913	

Dependent variable: no. of *bad mental health days* in 30 days before interview. Stigma in health care=1 if answer to the question “When seeking health care past 12 months, was experience worse, same, better than people of other races?” is “Worse than other races” and 0 if “Same as other races” or “Better than other races.” All estimates are weighted by the weights provided by the BRFSS. Regression diagnostics were conducted to identify multicollinearity using a VIF to indicate a potential problem. All VIF values were less than 2.1.

^***^ *p*<0.01, ^**^p<0.05, ^*^p<0.1.

CI, confidence interval; Coeff., coefficients from linear ordinary least square regressions.

**Table 4.** Associations of Perceived Stigma with Depressive Disorder Diagnosis–Logistic Regressions Using Behavioral Risk Factor Surveillance System Data 2012–2014

Variables	(1)	(2)	(3)	(4)
	OR	95% CI	AOR	95% CI
Stigma in health care	2.18^***^	1.83 to 2.60	1.61^***^	1.29 to 2.00
Female			1.77^***^	1.60 to 1.95
Race/ethnicity (Ref: White)				
African American			0.31^***^	0.27 to 0.37
Hispanic			0.56^***^	0.40 to 0.79
Asian			0.65^***^	0.54 to 0.78
Other			0.65^**^	0.44 to 0.95
Age (Ref: 18–24)				
25–34			1.71^***^	1.35 to 2.17
35–44			1.92^***^	1.52 to 2.41
45–54			1.86^***^	1.49 to 2.31
55–64			1.58^***^	1.28 to 1.96
65 or older			0.73^***^	0.59 to 0.90
Married			0.77^***^	0.69 to 0.85
College graduate			1.07	0.97 to 1.18
Employed			0.53^***^	0.47 to 0.60
Has health care coverage			1.39^***^	1.17 to 1.67
Could not see doctor because of cost			2.12^***^	1.86 to 2.42
Has a primary care physician			1.44^***^	1.25 to 1.65
Income (Ref: less than $10,000)				
$10,000 to less than $15,000			0.77^*^	0.58 to 1.02
$15,000 to less than $20,000			0.55^***^	0.42 to 0.72
$20,000 to less than $25,000			0.54^***^	0.42 to 0.70
$25,000 to less than $35,000			0.43^***^	0.33 to 0.57
$35,000 to less than $50,000			0.40^***^	0.31 to 0.52
$50,000 to less than $75,000			0.37^***^	0.28 to 0.49
$75,000 or more			0.28^***^	0.21 to 0.37
Constant	0.24^***^	0.23 to 0.25	0.29^***^	0.21 to 0.40
Observations	61,130		42,242	

Dependent variable:=1 if ever told that respondent had a depressive disorder. Stigma in health care=1 if answer to the question “When seeking health care past 12 months, was experience worse, same, better than people of other races?” is “Worse than other races” and 0 if “Same as other races” or “Better than other races.” Models in this table are estimated by logistic regression methods. All estimates are weighted by the weights provided by the BRFSS. Regression diagnostics were conducted to identify multicollinearity using a VIF to indicate a potential problem. All VIF values were less than 2.1.

^***^ *p*<0.01, ^**^p<0.05, ^*^p<0.1.

AOR, adjusted odds ratio; CI, confidence interval; OR, odds ratio.

Column [1] shows that the bivariate relationship is strongly significant. However, even after controlling for the confounding variables, stigma is still significantly associated with poor physical health days (*β*=2.79, confidence interval [CI]=1.84–3.75). The results suggest that the effect of stigma is large—individuals reporting stigma in health care setting are likely to have 2.79 additional poor health days during the 30 days before the interview. Table 3 estimates the same models, but for mental health, where the dependent variable is the number of days the respondent reported poor mental health during the 30 days before the interview. Key results are similar to what was found in the physical health analysis with one exception. The magnitudes of coefficients are higher in case of mental health suggesting that perceived stigma in health care settings has a stronger negative effect on mental health. Specifically, even after controlling for confounding factors, perceived stigma in health care settings is associated with 2.92 more days of poor mental health days reported (*β*=2.92, CI=1.97–3.86).

Table 4 presents estimates from models where the (binary) dependent variable is self-reported diagnosis for depressive disorder. This model is estimated by logistic regressions. According to the unadjusted odds-ratios, those reporting stigma in health care settings have significantly higher odds of being diagnosed with depressive disorder. After controlling for confounding variables in column [2], the adjusted odds ratio (AOR) drops, but remains significant. Specifically, perceived stigma in health care settings is associated with 61% higher odds of being diagnosed with depressive disorder (AOR=1.61, CI=1.29–2.00) compared to the respondents who did not perceive such stigma.

In all regression tables, most control variables are statistically significant. Women report a higher number of poor mental health days than men, but the pattern is reversed for physical health days. Older respondents report bad physical and mental health days at significantly higher rates than younger respondents. Individuals, who are married, belong to higher income groups, have college degrees, and are employed report significantly fewer numbers of bad physical and mental health days. One notable finding was that once we control for stigma, minority groups reported fewer days of poor physical and mental health compared to the referent white group.

## Discussion

We tested and found support for the hypothesis that stigma, attributed to being a racial or ethnic minority, in health care settings is associated with poorer physical health and worse mental health. Higher number of days of poor physical health and poor mental health were associated with perceived stigma in health care. In addition, although most respondents, regardless of race or ethnicity, had health insurance coverage, a notable proportion still found health care too costly (7–15%). The highest levels of perceived costliness were in people of color. A similar trend was found related to respondents having a primary care physician, which has been shown to improve health (particularly if the physician is based in a patient-centered medical home).^36,37^ Only 63% of Asian respondents (75% African American and 70% Hispanic) had a designated primary care provider compared with 80% of whites in our sample. This is particularly worrisome, considering the past studies that have found that African Americans and Hispanics have lesser access to health care in general, and now our study reinforces this finding with evidence of limited access not only to health care facilities but also to having a primary care provider.^38.39^ We also found that whites did not have the lowest average days of poor physical and mental health. Asians Americans had the lowest rates at 2.05 poor physical health days in the past month and 2.14 poor mental health days in the past month. Previous studies on the Healthy Migrant Effect cite the potential that immigrant populations are generally healthier, due to a selection bias wherein only the heartiest individuals emigrate.^5,13,14^ Considering the preponderance of foreign-born persons within the Asian classification,^40^ it is possible that Asian Americans' good health status may reflect a combination of personal experiences, race and ethnicity, and nativity. This provides additional support for the idea that the sociocultural experiences (e.g., experiences with stigma) of minority Americans may influence health and well-being and that race and ethnicity, in and of itself, is not the sole associate of good health. In summary, the results show that perceived stigma in health care settings is strongly associated with higher odds of depression and poorer health independent of demographic factors.

### Limitations

Limitations should be considered when applying or extending these findings. First, the BRFSS relies on self-report data from the respondent.^35^ Self-reported data that are not corroborated by clinical or work records are subjected to a number of sources of possible errors and biases (e.g., desirability bias, recall bias/response error). There is also the possibility that respondents were different from those who declined to participate. BRFSS interviews were only available in English and Spanish, so willing respondents who did not speak English or Spanish were excluded.^35^ Specific to our study, the outcome measures of the number of poor health days have limitations. For example, apart from being a subjective measure, the number of poor health days was only reported for the previous 30 days; thus, it does not capture long-term behavior. Finally, causality cannot be inferred from cross-sectional data.

## Conclusion

We find support for the importance of reducing stigma in health care settings to improve physical and mental health outcomes in people of color. Stigma reduction in health care settings could lead to the creation of more diversity-friendly environments that, in turn, could then result in fewer days lost to poor physical and mental health. Reducing days of poor health would not only positively influence the health care system by reducing utilization but could also improve economic productivity by increasing days of good health. Thus, eradicating stigma against people of color is not only a social good but also could have direct and indirect positive impacts on the health care and employment sectors of the wider community. However, to do so thoughtfully, public health researchers and social scientists should examine the persistent effects of institutionalized and structural stigma (e.g., stigma in health care, barriers to people of color entering medical school limiting the number of diverse clinical providers) that relate to and predate Jim Crow. Future studies should measure and apply different forms of stigma assessing individual and compound effects across a more specified range of health conditions.

## Abbreviations Used

AOR: adjusted odds ratio
BRFSS: Behavioral Risk Factor Surveillance System
CI: confidence interval
OR: odds ratio
VIF: variance inflation factor

## References

[1] Alba R, Barbosa GY Room at the top? Minority mobility and the transition to demographic diversity in the USA. Ethnic Racial Stud. 2016;39:917–938

[2] United States Census Bureau. Quick Facts United States. 2016. Available at https://www.census.gov/quickfacts/fact/table/US/PST045216 Accessed 120, 2018

[3] Collins RL, Haas A, Haviland AM, et al. What matters most to whom: racial, ethnic, and language differences in the health care experiences most important to patients. Med Care. 2017;55:940–947 28930888 10.1097/MLR.0000000000000804

[4] Raymond-Flesch M, Siemons R, Pourat N, et al. There is no help out there and if there is, it's really hard to find: a qualitative study of the health concerns and health care access of Latino DREAMers. J Adolesc Health. 2014;55:323–328 25151054 10.1016/j.jadohealth.2014.05.012

[5] Lopez-Jimenez F, Lavie CJ Hispanics and cardiovascular health and the hispanic paradox: what is known and what needs to be discovered? Progr Cardiovasc Dis. 2014;57:227–229 10.1016/j.pcad.2014.09.007 25269065

[6] George S, Duran N, Norris K A systematic review of barriers and facilitators to minority research participation among African Americans, Latinos, Asian Americans, and Pacific Islanders. Am J Public Health. 2014;104:e16–e31 10.2105/AJPH.2013.301706 PMC3935672 24328648

[7] Eaton LA, Driffin DD, Kegler C, et al. The role of stigma and medical mistrust in the routine health care engagement of black men who have sex with men. Am J Public Health. 2015;105: e75–e82 10.2105/AJPH.2014.302322 PMC4318301 25521875

[8] Major B, Dovidio JF The Oxford Handbook of Stigma, Discrimination, and Health. Oxford, United Kingdom: Oxford University Press, 2017

[9] De Luca SM, Blosnich JR, Hentschel EAW, et al. Mental health care utilization: how race, ethnicity and veteran status are associated with seeking help. Commun Mental Health J. 2016;52:174–179 10.1007/s10597-015-9964-3 26659853

[10] Goffman E. Stigma; Notes on the Management of Spoiled Identity. Englewood Cliffs, NJ: Prentice-Hall, 1963

[11] Turan B, Budhwani H, Browning WR, et al. How does stigma affect people living with HIV? The mediating roles of internalized and anticipated HIV stigma in the effects of perceived community stigma on health and psychosocial outcomes. AIDS Behav. 2017;21:283–291 27272742 10.1007/s10461-016-1451-5 PMC5143223

[12] Fazelia P, Turan JM, Budhwani H, et al. Moment-to-moment within-person associations between acts of discrimination and internalized stigma in people living with HIV: an experience sampling study. Stigma Health. 2017;2:216–228 28966982 10.1037/sah0000051 PMC5614514

[13] Budhwani H, Hearld KR, Chavez-Yenter D Depression disparities in racial and ethnic minorities: the impact of nativity and discrimination. J Racial Ethn Health Disparities. 2015;2:34–42 26863239 10.1007/s40615-014-0045-z

[14] Hearld KR, Budhwani H, Chavez-Yenter D Panic attacks in minority Americans: the effects of alcohol abuse, tobacco smoking, and discrimination. J Affect Disord. 2015;174:106–112 25496758 10.1016/j.jad.2014.11.041

[15] Budhwani H, Hearld KR, Milner A, et al. Transgender Women's Experiences with Stigma, Trauma, and Attempted Suicide in the Dominican Republic. Suicide Life Threat Behav. 2018;48:788–796 28950402 10.1111/sltb.12400

[16] Kim G, Parmelee P, Bryant AN, et al. Geographic region matters in the relation between perceived racial discrimination and psychiatric disorders among black older adults. Gerontologist. 2017;57:1142–1147 27927726 10.1093/geront/gnw129 PMC5881795

[17] Odem ME. Immigration politics in the New Latino South. J Am Ethn Hist. 2016;35:87–91

[18] Hausmann LRM, Jeong K, Bost JE, et al. Perceived discrimination in health care and health status in a racially diverse sample. Med Care. 2008;46:905–914 18725844 10.1097/MLR.0b013e3181792562 PMC3424509

[19] Gee GC. A multilevel analysis of the relationship between institutional and individual racial discrimination and health status. Am J Public Health. 2002;92:615–623 11919062 10.2105/ajph.92.4.615 PMC1447127

[20] Kwate NO, Valdimarsdottir HB, Guevarra JS, et al. Experiences of racist events are associated with negative health consequences for African American women. J Natl Med Assoc. 2003;95:450–460 12856911 PMC2594553

[21] Wagner J, Abbott G Depression and depression care in diabetes: relationship to perceived discrimination in African Americans. Diabetes Care. 2007;30:364–366 17259510 10.2337/dc06-1756

[22] Borrell LN, Kiefe CI, Williams DR, et al. Self-reported health, perceived racial discrimination, and skin color in African Americans in the CARDIA study. Soc Sci Med. 2006;63:1415–1427 16750286 10.1016/j.socscimed.2006.04.008

[23] Ryan AM, Gee GC, Laflamme DF The Association between self-reported discrimination, physical health and blood pressure: findings from African Americans, Black immigrants, and Latino immigrants in New Hampshire. J Health Care Poor Underserved. 2006;17:116–132 10.1353/hpu.2006.0092 16809879

[24] Borrell LN, Jacobs DR Jr, Williams DR, et al. Self-reported racial discrimination and substance use in the Coronary Artery Risk Development in Adults Study.Am J Epidemiol. 2007 10.1093/aje/kwm180 17698506

[25] Thornicroft G, Mehta N, Clement S, et al. Evidence for effective interventions to reduce mental-health-related stigma and discrimination. Lancet. 2016;387:1123–1132 26410341 10.1016/S0140-6736(15)00298-6

[26] MacDonald S, Hausmann LRM Sileanu FE, et al. Associations between perceived race-based discrimination and contraceptive use among Women Veterans in the ECUUN Study. Med Care. 2017;55:S43–S49 28806365 10.1097/MLR.0000000000000746 PMC5605763

[27] Bleser WK, Miranda PY, Jean-Jacques M Racial/ethnic disparities in influenza vaccination of chronically ill US adults: the mediating role of perceived discrimination in health care. Med Care. 2016;54:570–577 27172536 10.1097/MLR.0000000000000544 PMC6060271

[28] Richardson MP, Waring ME, Wang ML, et al. Weight-based discrimination and medication adherence among low-income African Americans with hypertension: how much of the association is mediated by self-efficacy? Ethn Dis. 2014;24:162–168 24804361

[29] Bowleg L. When black + lesbian + woman≠black lesbian woman: the methodological challenges of qualitative and quantitative intersectionality research. Sex Roles. 2008;59:312–325

[30] Shields SA. Gender: an intersectionality perspective. Sex Roles. 2008;59:301–311

[31] Purdie-Vaughns V. Intersectional invisibility: the distinctive advantages and disadvantages of multiple subordinate-group identities. Sex Roles. 2008;59:377–391

[32] Collins PY, von Unger H, Armbrister A Church ladies, good girls, and locas: stigma and the intersection of gender, ethnicity, mental illness, and sexuality in relation to HIV risk. Soc Sci Med. 2008;67:389–397 18423828 10.1016/j.socscimed.2008.03.013 PMC2587415

[33] Waldron ME, Brooks HTL Marriage protection and marriage selection—prospective evidence for reciprocal effects of marital status and health. Soc Sci Med. 1996;43:113–123 8816016 10.1016/0277-9536(95)00347-9

[34] Hearld KR, Badham A, Budhwani H Statistical effects of religious participation and marriage on substance use and abuse in racial and ethnic minorities. J Rel Health. 2017;56:1155–1169 10.1007/s10943-016-0330-8 27900640

[35] Centers for Disease Control and Prevention (CDC). Behavioral Risk Factor Surveillance System (BRFSS). 2017. Available at https://www.cdc.gov/brfss/index.html Accessed on 120, 2018

[36] Hearld KR, Hearld LR, Landry AY, et al. Evidence that patient centered medical homes are effective in reducing Emergency Department (ED) Admissions for patients with depression. Health Serv Manage Res. 2019;32:26–35 30149725 10.1177/0951484818794340

[37] Hearld L, Hearld KR, Budhwani H Bridging the gap: the role of the patient-centered medical home in reducing time to follow-up after hospital discharge. J Ambul Care Manage. 2016;39:209–219 27232682 10.1097/JAC.0000000000000104

[38] Mertz EA, Wides CD, Kottek AM, et al. Underrepresented minority dentists: quantifying their numbers and characterizing the communities they serve. Health Affairs. 2016;35:2190–2199 27920306 10.1377/hlthaff.2016.1122 PMC5364808

[39] Garces LM, Mickey-Pabello D Racial diversity in the medical profession: the impact of affirmative action bans on underrepresented student of color matriculation in medical schools. J Higher Educ. 2015;86:264–294 26052161 10.1353/jhe.2015.0009 PMC4454423

[40] Budhwani H, De P Human papillomavirus vaccine initiation in Asian Indians and Asian subpopulations: a case for examining disaggregated data in public health research. Public Health. 2017;153:111–117 29028565 10.1016/j.puhe.2017.07.036

